# Effect of different protein sources on satiation and short-term satiety when consumed as a starter

**DOI:** 10.1186/1475-2891-10-139

**Published:** 2011-12-23

**Authors:** Rania Abou-Samra, Lian Keersmaekers, Dino Brienza, Rajat Mukherjee, Katherine Macé

**Affiliations:** 1 Nestlé Research Center, Nestec Ltd, Lausanne, Switzerland; 2 Department of Human Biology, Nutrim, FHML, Maastricht University; P O Box 616, 6200 MD Maastricht, Netherlands; 3 Current address: Atrium Medical Center, Heerlen, Netherlands

## Abstract

**Background:**

Because the source of protein may play a role in its satiating effect, we investigated the effect of different proteins on satiation and short-term satiety.

**Methods:**

Two randomized single-blind cross-over studies were completed. In the first study, we investigated the effect of a preload containing 20 g of casein, whey, pea protein, egg albumin or maltodextrin vs. water control on food intake 30 min later in 32 male volunteers (25 ± 4 yrs, BMI 24 ± 0.4 kg/m^2^). Subjective appetite was assessed using visual analogue scales at 10 min intervals after the preload. Capillary blood glucose was measured every 30 min during 2 hrs before and after the ad libitum meal. In the second study, we compared the effect of 20 g of casein, pea protein or whey vs. water control on satiation in 32 male volunteers (25 ± 0.6 yrs, BMI 24 ± 0.5 kg/m^2^). The preload was consumed as a starter during an ad libitum meal and food intake was measured. The preloads in both studies were in the form of a beverage.

**Results:**

In the first study, food intake was significantly lower only after casein and pea protein compared to water control (P = 0.02; 0.04 respectively). Caloric compensation was 110, 103, 62, 56 and 51% after casein, pea protein, whey, albumin and maltodextrin, respectively. Feelings of satiety were significantly higher after casein and pea protein compared to other preloads (P < 0.05). Blood glucose response to the meal was significantly lower when whey protein was consumed as a preload compared to other groups (P < 0.001). In the second study, results showed no difference between preloads on ad libitum intake. Total intake was significantly higher after caloric preloads compared to water control (P < 0.05).

**Conclusion:**

Casein and pea protein showed a stronger effect on food intake compared to whey when consumed as a preload. However, consuming the protein preload as a starter of a meal decreased its impact on food intake as opposed to consuming it 30 min before the meal.

## Background

The rise in global obesity prevalence in both adults and children may lead to a decrease in life expectancy [1]. Accordingly, there is an urgent need to find solutions to help control the rise in obesity. Protein intake is associated with weight loss [2]. This effect has been attributed to the greater satiating potential of proteins compared to other macronutrients [3,4].

The source of protein may play a role in its satiating effect; however, inconsistent data exists from human studies. Milk proteins have been considered to increase satiety and suppress short-term food intake compared to other sources [5], but the contribution of complete milk proteins vs. whey protein or casein is still not clear. Whey protein showed a stronger suppression of hunger [6] and lower food intake [7] compared to casein. However, other studies have found similar effects on satiety and food intake between whey protein and casein [8,9]. Eggs are a good source of proteins. Recently, eggs have been shown to enhance satiety and decrease energy intake when consumed for breakfast [10] resulting in higher weight loss during energy restriction [11]. There is limited and inconsistent evidence on the effect of egg proteins on appetite regulation. Studies have found either the same effect compared to gelatine, casein, soy, pea or wheat protein [12] or lower effect on satiety and short-term food intake compared to whey and soy protein [13]. Currently proteins of plant origin are gaining interest as an alternative to animal proteins, favoured by consumers shifting away from animal-derived proteins for health and environmental reasons. One recent study investigated pea protein and showed stronger suppression of appetite compared to whey protein when 15 g of pea protein hydrolysate was consumed in overweight subjects [14]; however, evidence remains limited.

Dose plays an important role on the duration of effect of proteins on food intake. It is clear that around 50 g of protein in a food or a meal has a strong effect on satiety [15]. Nevertheless, the application of such a dose in food products remains limited. Interestingly, a recent dose-response study has shown that 20 g of whey protein is able to suppress food intake 30 min later [16].

The association of protein with satiation is not known. Only one study showed that subjects consumed less from a high protein omelette compared to a high fat omelette consumed ad libitum [17]. Satiation develops during a meal and results in the termination of a meal while satiety develops after a meal and inhibits further eating [17,18]. To date, most of the literature has dealt with satiety and little attention was given to satiation.

In this context, we investigated the satiety benefits of 20 g of different protein sources on a meal consumed either immediately after the preload or 30 min later.

## Methods

### Experiment 1

#### Subjects

Thirty-two healthy male volunteers were recruited from the local vicinity. Subjects were included if they were 20-35 years (yrs) old, healthy (determined by a medical questionnaire), with a Body Mass Index (BMI) between 20 and 27 kg/m^2^, scoring 8 or below on the disinhibition score, and one of the following: 11 or below on the dietary restraint score; or 7 or below, for the susceptibility to hunger score in the "Three factor eating questionnaire" (TFEQ)[19], willing to consume no alcohol and not smoke on the evening before the test, eating breakfast regularly, willing to eat all the foods served in the study, performing a maximum of 10 hours of intense physical activity per week, able to commit to the duration of the study.

All subjects gave their written informed consent before the start of the study. The study protocol was approved by the Ethical Committee of the Université De Lausanne in accordance with the Helsinki declaration (15/08 # 3).

#### Study design

The study was an open, single-blind randomized, cross-over trial. Eligible subjects participated in a total of 7 sessions including 1 training session and 6 test sessions. Test sessions were scheduled at least 2 days apart in order to minimize taste fatigue related to the ad libitum meals. To minimize variability, subjects were asked to keep their evening meals and activity levels on the day before the test as similar as possible and to refrain from drinking alcohol on the evening before the test. Subjects were also asked not to eat or drink anything except non-carbonated water after 21h00.

#### Test foods

Six preloads were tested in this experiment. Pea protein, casein, whey, maltodextrin, egg albumin and a water control. The protein preloads included 20 g of egg albumin, casein protein, whey protein, pea protein and maltodextrin dissolved in non-carbonated water. The control preload was 250 ml of non-carbonated water. All the preloads except for the water control contained around 80 kcal (20 g protein) and were adjusted to a total volume of 200 ml (Table 1). The water control treatment was adjusted to 250 ml in order to match the volume of the test preload (200 ml) plus the volume of water used to rinse their mouth (50 ml) right after.

**Table 1:** Composition and nutrition information of the preloads served in Experiment 1 and 2

Food item	Wt (g)	Energy (kcal)	Protein (g)	Carbohydrate(g)	Sugars (g)	Fat (g)	Fiber (g)
**Casein protein^a^**	23	80	20	0.1	0.1	0.3	
**Egg albumin^b^**	24	94	20	1.1	-	-	-
**Pea protein^c^**	22.2	78	20	< 1	-	-	0.5
**Whey protein^d^**	22.2	83	20	< 1	-	-	-
**Maltodextrin^e^**	21	80	-	20	20	-	-
**Water^f^**	250	-	-	-	-	-	-

^a ^Hungarian Dairy Research Institute, 85% protein (micellar casein)

^b ^Ovobest, GmbH, 80% protein

^c ^Pisane F9, Cosucra-Groupe Warcoing S.A., 90% protein

^d ^Whey Protein Isolate Prolacta 90, BBA Lactalis, 90% protein

^e ^Cargill Houbourdin SAS, France, 96% maltodextrin

^f ^Vittel, Nestle

The amount of protein and maltodextrin powder added to the preloads was adjusted according to the protein and maltodextrin content of the powders. Maltodextrin was included as a positive or caloric control to investigate if the protein effect on food intake and satiety is due to its caloric content. Aspartame as well as aromas and citric acid were added to improve the taste of the preloads (Table 2). The amount of aspartame was adjusted to have equal sweetness in all the protein and maltodextrin preloads. The preloads were served 30 minutes before an ad libitum meal prepared at the experimental kitchen.

**Table 2:** Recipe of test preloads served in Experiment 1

	Whey	Maltodextrin	Pea	Casein	Egg Albumin
**Dosage (g)**	22.2	21	22.2	23.4	24

**Water (g)**	200	200	200	200	200

**Aroma** **(ml)**	Lemon 0.16	Lemon 0.16	Lemon 0.2	Vanilla 0.1	Coffee 0.2

**Aspartame (g)**	0.1	0.05	0.1	0.1	0.1

**Citric Acid (g)**	0.32	0.16	0.4	-	-

The ad libitum meal was a "Crème Budwig", which is a typical Swiss breakfast comprised of a combination of cereals, quark, nuts and fruits. Energy and macronutrient content on 100 g of the ad libitum breakfast was 112 kcal, 5.7 g protein, 17.4 g carbohydrate (CHO), 2.5 g fat and 2 g fiber respectively. Subjects were allowed to eat as much or as little as they want from the served foods but were not allowed to take food with them to consume later. The ad libitum meal was served in excess to allow subjects to eat until comfortably full. Subjects chose between 2 types of Crème Budwig (apple-orange or pear-kiwi) but were asked to consume the same type at each session. Meals were accompanied by non-carbonated water (unlimited amount).

#### Outcomes

##### Energy intake

Energy intake was measured by calculating the amount of food consumed from the ad libitum meal. All foods and water were pre-weighed by the investigators before serving and left-overs were weighed afterwards to calculate the amounts eaten. The food tray was served in the Evaluation Room and subjects were able to eat while seated in individual cubicles.

##### Visual analogue scales (VAS) ratings

VAS ratings were collected before and after the preload and at 10 min intervals between the preload and the ad libitum meal. A validated electronic system based on a Dell Pocket PC was used [20]. Subjects rated their motivation-to-eat and other sensations on a horizontal non-graded, unlabelled line anchored at each end by an opposed statement (e.g. "not at all hungry", "as hungry as I ever felt"). In the Pocket PC system, the subjects answered the questions on a 70 mm VAS by clicking on the screen with the aid of a plastic marker. The computer measures the distance in mm from the left end of the scale to the point where the subject has inserted a line. An automatic computation is made to normalize this distance to 100 mm (standard distance). All entries are automatically timed and dated. The motivation-to-eat questions were based on Hill and Blundell's motivation-to-eat questionnaire [21]. A French translation of the following questions was used:

"How strong is your desire to eat?" (very weak - very strong)

"How hungry do you feel?" (not hungry at all - as hungry as I ever felt)

"How full do you feel?" (not full at all - very full)

"How thirsty do you feel?" (not at all thirsty - very thirsty)

"How much do you think you could eat?" (nothing at all - a large amount)

To assess liking or palatability, the following question was asked twice, once after consuming each preload and once after consuming the ad libitum meal:

"How much did you like this food or beverage?" (not at all - very much)

To assess the sweetness of the preloads, the following question was asked after the preload:

"How sweet was the beverage?" (very sweet - not sweet at all)

##### Capillary blood glucose

Capillary blood glucose was measured using a glucometer (Accu-check Compact Plus) and a lancet device (Soft Clix, Roche Diagnostics). A correction factor of 1.12 is programmed into the monitor to convert the results from capillary blood to venous plasma [22]. Thus, the reported results correspond to plasma glucose concentrations. The total amount of blood taken over the study was about 1\2 a teaspoon. The proper use of a finger prick blood sampler (lancet) and glucose monitor was explained to the study participants during the training session. The measurement of the capillary glucose was supervised by the investigators. To control for CV of the glucometer, the glucose measures were performed in duplicates and the average of the 2 measures was calculated. In instances where the difference between the 2 measures was equal or above to 10%, a third measure was done and the average of the 3 was calculated.

##### General procedure

**Figure 1:**
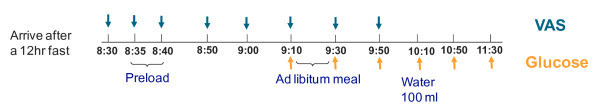
**Test session timeline for Experiment 1**. Subjects arrived at 8h15 in the morning and completed a baseline questionnaire to assess their state of well being and whether they were fasted on the day of the session. They then completed motivation-to-eat ratings. At 8h35 subjects consumed the preload within 5 min accompanied by 50 ml of water or just the water preload (250 ml), and rated their satiety feelings. Between 8h40 and 9h10, subjects continued to complete satiety ratings. At 9h10, subjects measured their plasma blood glucose after they completed the satiety ratings questions. Immediately after, they consumed an ad libitum meal. Once finished (9h30), the subjects completed 2 further sets of satiety questionnaires. They also measured their plasma glucose at different time-intervals.

Subjects came to the Nestlé Research Centre for 7 sessions, including one training session and 6 test sessions. During the training session, subjects' weight and height was measured and they received a letter with information about the study. They were introduced to the electronic VAS pocket PCs. Subjects tasted the beverages and foods used in the study and chose one of the two breakfasts that they preferred. They answered medical and dietary questionnaires and a French translation of the TFEQ [19]. Subjects were told that they were not allowed to drink alcohol (alcohol increases passive over-consumption [23] or do vigorous exercise the day before the test day. Subjects were told that the aim of the study was to evaluate the properties of different protein beverages with no mention of food intake measurement. The test sessions timeline is shown in Figure 1.

On the day of the test, subjects arrived at 8h15 in the morning at the Nestlé Research Center. They were invited to go into the Evaluation Room and sit in individual cubicles. Subjects were asked to refrain from talking, surfing the internet or using mobile phones, except for an emergency while they answer VAS scales or while consuming the preload or ad libitum meal. Subjects completed a baseline questionnaire to assess their state of well being and whether they were fasted on the day of the session. They then completed motivation-to-eat ratings. At 8h35 subjects consumed the preload within 5 min accompanied by 50 ml of water or just the water preload (250 ml), and rated their satiety feelings on Pocket PCs provided by the investigators. Between 8h40 and 9h10, subjects continued to complete satiety ratings on their Pocket PCs, as prompted by an alarm. At 9h10, subjects measured their plasma blood glucose after they completed the satiety ratings questions. Immediately after, they consumed an ad libitum meal. No other foods or beverages were allowed during the test session. Once finished (9h30), the subjects completed 2 further sets of satiety questionnaires on their Pocket PC. They also measured their plasma glucose at different time-intervals (6 times till 11h30).

### Experiment 2

#### Subjects

Thirty-two healthy male volunteers were recruited from the local vicinity. Most of the subjects from experiment 1 also participated in experiment 2 except for 3 subjects. All subjects were screened for the same inclusion criteria as experiment 1. All subjects gave their written informed consent before the start of the study. The study protocol was approved by the Ethical Committee of the Université De Lausanne in accordance with the Helsinki declaration (15/08 #5).

#### Study design

The study was an open, singly-blind, randomized, cross-over trial. Eligible subjects participated in a total of 5 sessions including 1 training session and 4 test sessions. Study design details were similar to Experiment 1.

#### Test foods

Four preloads were tested in this experiment. Whey protein, casein protein, pea protein and water control. We decided to only use protein preloads for experiment 2 as the objective is to confirm the observed effect of pea protein and casein and to compare it with another protein that did not show an effect on food intake. Whey was chosen due to its reported effect on food intake in the literature. The protein preloads included 20 g of casein protein, whey protein or pea protein dissolved in 250 ml non-carbonated water. The control preload was 250 ml of non-carbonated water. The water control treatment was adjusted to 250 ml in order to match the volume of the test preload (200 ml) plus the volume of water used to rinse their mouth (50 ml) right after. All the preloads except for the water control contained around 80 kcal (20 g protein) (Table 1). In this experiment, the protein preloads were homogenized and the pH was adjusted to improve the taste and texture. Aspartame and aromas were added as well to improve the taste.

The ad libitum meal was a Bircher Muesli consisting of yoghurt and muesli. Energy and macronutrient content of 100 g of ad libitum meal was 160 kcal, 4.7 g protein, 14 g CHO, 2 g fat and 1.5 g fiber respectively. Subjects made a choice between three different yoghurts for the ad libitum breakfast. For every test session the same breakfast was served. Subjects were allowed to eat as much or as little as they want from the served foods but were not allowed to take foods with them to consume later. The ad libitum meal was served in excess to allow subjects to reach satiation. Meals were accompanied by non-carbonated water (unlimited amount).

#### Outcomes

##### Energy intake

Measurement of energy intake was similar to experiment 1.

##### Visual analogue scales

The method used to measure ratings for motivation-to-eat and palatability questions was similar to experiment 1. However, in the present experiment, the following questions were also asked between the preload and ad libitum meal to distract the subjects from thinking about the palatability of the preload while consuming the ad libitum meal:

"How happy do you feel?" (not happy at all - very happy)

"How stressed do you feel?" (not stressed at all - very stressed)

"Did you sleep well last night?" (not well at all - very well)

"How tired do you feel?" (not tired at all- very tired)

##### General procedure

Subjects came to the Nestlé Research Centre for 5 sessions, including a training session and 4 test sessions. The training session was described in Experiment 1, General Procedure section.

On the day of the test, subjects arrived fasted (from 22 h the evening before) at 8.45 h. They were invited to go into the Evaluation Room and sit in individual cubicles. Before starting, subjects answered a questionnaire about their wellbeing and whether they were fasted on the day of the test. At 9.00 h subjects answered their first VAS questions (t = -20) and subsequently they received a preload. The order of the four preloads was randomized. Subjects were told to finish the drink within 5 minutes. They then answered a palatability question about the drink along with unrelated mood questions. Right after, the ad libitum meal was served. Subjects were allowed to eat as much as they liked. A bottle of water was offered with the breakfast. After breakfast VAS questions were answered at 0, 15, 30 and 45 minutes.

### Statistical analysis

Results are presented as mean ± standard error. Energy intake was analyzed using a mixed model with a random subjects effect (to take into account the correlation between the repeat measurements for each subject) and fixed treatment effect. Baseline covariates were adjusted for. Multiple pair-wise treatment comparisons were carried out using Tukey's Honest Significant Difference procedure. The secondary outcome measures were analyzed similarly within the mixed model analysis of covariance framework. The incremental area under the curve (AUC) was calculated using the trapezoide rule. Suitable normalizing transforms were applied to certain incremental AUC measures. In the case were the normalizing transformation failed, non-parametric methods were used.

Sample size was calculated based on the expected difference of 75% of 80 kcal (provided by the treatment preloads) i.e. 60 kcal in energy intake between a control treatment and the test treatment. The within subject standard deviation is estimated to be 79 kcal from previous trials. With these estimates, 30 subjects would be enough to test the difference between any one of the test preloads against water with 80% power at 5% level of significance. The sample size calculation was performed based on data from our previous study published as an abstract [24].

A combined satiety score (CSS) was calculated as composite satiety score using the measures for fullness, desire to eat, hunger and prospective food consumption (PFC) =

[Fullness+(100-Desiretoeat)+(100-Hunger)+(100-PFC)]∕4.

The formula reflected the 4 questions on the motivation-to-eat questionnaire.

The range for the CSS is between 0 and 100, 0 indicating maximum appetite sensations and 100 minimum appetite sensations. This score is based on the concept that the 4 motivational ratings, the inverse for hunger, the inverse for desire to eat, the inverse for PFC and fullness can account for an overall measure of satiety [25,26].

Percent energy compensation was calculated as

$$\text{Compensation }\left(\%\right)=\left[\text{Energy intake after water }\left(\text{kcal}\right)\;-\text{ Energy intake after test food }\left(\text{kcal}\right)\right]\text{/ test food energy content }\left(\text{kcal}\right)\times 100$$

It provides a measure of the percentage reduction in energy intake at the next meal due to the test food calories. This reduction is relative to the energy intake after the water control [27]. Caloric compensation of < 100% indicated that the subject had low compensation for the preload energy at the test meal, whereas a score > 100% indicates overcompensation for the preload energy at the test meal.

## Results

### Experiment 1

Thirty-two male subjects were recruited for the study. One subject dropped out and another was excluded since he did not like to have breakfast and was not feeling hungry in the morning (exclusion criteria). Another subject did not complete the study since he underwent a leg surgery. Twenty-nine subjects completed the experiment with a mean age of 25 ± 4 yrs and a mean BMI of 24 ± 0.4 kg/m^2^.

**Figure 2:**
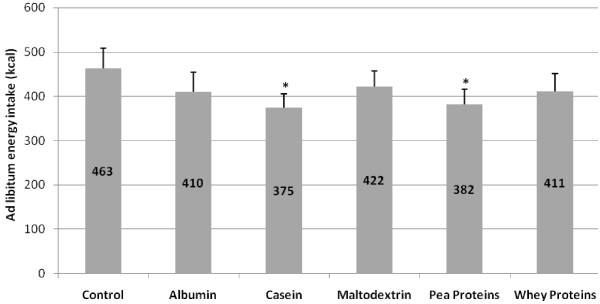
**Energy intake (Mean + SEM) from the ad libitum meal 30 min after the preload**. The respective means are embedded in the columns. *Significantly different from water at P < 0.05. Energy intake from the ad libitum meal was significantly lower after the casein and pea protein preloads compared to water control (P = 0.02; 0.04 respectively).

Energy intake from the ad libitum meal was significantly lower after the casein and pea protein preloads compared to water control (P = 0.02; 0.04 respectively). There was no significant difference among the other preloads (Figure 2). Water intake during the ad libitum meal did not differ between the treatments. Caloric compensation was 110, 103, 62, 56 and 51% after casein, pea protein, whey, albumin and maltodextrin, respectively. Cumulative energy intake, calculated as the sum of calories from the preload and the ad libitum meal, was not significantly different among the treatments.

**Figure 3:**
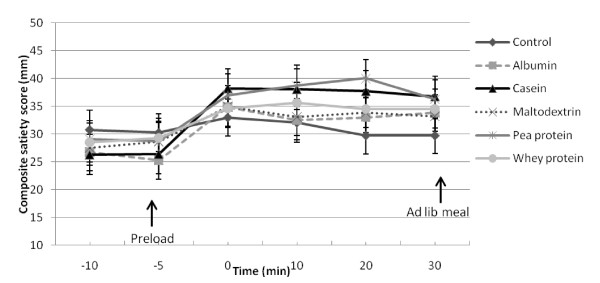
**Combined satiety score ratings (Mean ± SEM) before and after consumption of the preload**. The CSS ratings were significantly higher after the casein and pea protein preloads compared to the other preloads (P < 0.05). CSS was the lowest after the water control while the ratings were the same after albumin, whey and maltodextrin but higher than the water control (P < 0.05).

The CSS ratings were significantly higher after the casein and pea protein preloads compared to the other preloads (P < 0.05). CSS was the lowest after the water control while the ratings were the same after albumin, whey and maltodextrin but higher than the water control (Figure 3).

Pea protein preload had significantly lower palatability compared to whey, albumin, maltodextrin and water (P = 0.0001. Casein preload had similar palatability to pea protein, whey and albumin but lower than water and maltodextrin (P < 0.05). Scores were 18 ± 3.3, 45.2 ± 4.4, 40.2 ± 5, 65.9 ± 4.1, 68 ± 4.1 mm for pea protein, whey, albumin, maltodextrin and water respectively.

Both casein and pea protein preloads had significantly lower perceived sweetness compared to the other protein preloads and maltodextrin (P < 0.05). Sweetness did not differ between maltodextrin, whey and albumin preloads. Scores were 47.3 ± 3.6, 47.44 ± 3.6, 70.24 ± 3.3, 60.94 ± 3.3, 60.37 ± 3.1, 9.11 ± 2.3 mm for casein, pea protein, albumin, whey, maltodextrin and water respectively.

Feelings of thirst were significantly lower after the water control compared to the other preloads (P < 0.05), but there was no difference between the caloric preloads.

**Figure 4:**
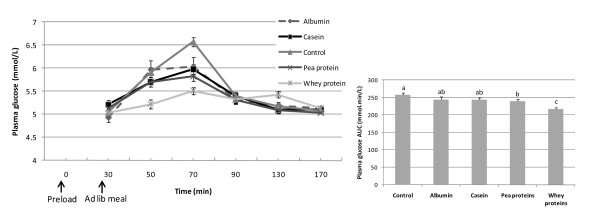
**Left: plasma glucose response to the ad libitum meal consumed 30 min after the preload (Mean ± SEM). Right: plasma glucose response area under the curve (AUC) (Mean + SEM)**. Bars with different superscripts are significantly different at P < 0.05. The response after the maltodextrin preload was not included in the analysis since it is 100% carbohydrate and resulted in a pre-meal baseline value that was much higher and incomparable to the protein and control values. Plasma glucose response to the ad libitum meal was significantly lower when whey protein was consumed as a preload compared to the other preloads (P < 0.001).

Plasma glucose response to the ad libitum meal was significantly lower when whey protein was consumed as a preload compared to the other preloads (P < 0.001). This was further observed in the incremental AUC, where plasma glucose AUC was significantly lower after the whey preload compared to the other preloads (P < 0.001) (Figure 4).

### Experiment 2

Thirty-two male subjects were recruited. One subject dropped out and 31 subjects completed all the sessions. Subjects had a mean age of 25 ± 0.63 yrs and mean BMI of 24 ± 0.5 kg/m^2^.

**Figure 5:**
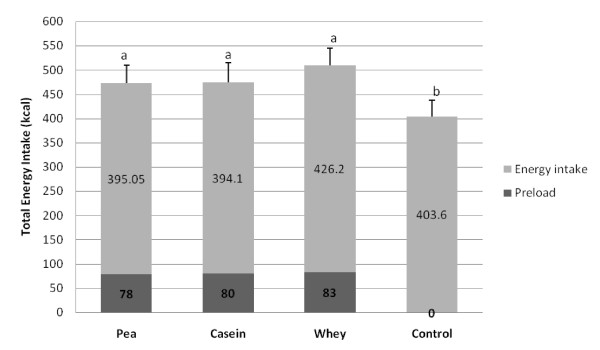
**Cumulative energy intake (Mean + SEM) from both the preload and ad libitum meal**. The respective means are embedded in the columns. Bars with different superscripts are significantly different at P < 0.05. Cumulative energy intake, calculated as the preload + the ad libitum meal, was significantly higher after all three protein preloads compared to the water control (P < 0.05).

Cumulative energy intake, calculated as the preload + the ad libitum meal, was significantly higher after all three protein preloads compared to the water control (P < 0.05) (Figure 5). Ad libitum energy intake (without the preload calories) was not significantly different between all preloads including the water control. Caloric compensation during the ad libitum meal of the pea, casein and whey preloads was 10.9, 11.8 and -27.3% respectively. The negative compensation after the whey preload indicates that energy intake was higher than after the water control. Water intake during the ad libitum meal was not significantly different between the different preloads.

The CSS ratings were adjusted for the baseline rating (t = 0 min) and showed an increase after consumption of the preload and ad libitum meal that was sustained until 45 min later, the end of the study session. There were no significant differences between all 4 different preloads.

When palatability was measured right after the consumption of the preload, pea protein had significantly lower palatability compared to whey and control preloads (P < 0.05). Palatability after casein, whey and control preloads did not differ. However, when palatability was assessed after the ad libitum meal, the statistical difference between the preloads disappeared.

## Discussion

We have shown that 20 g of casein or pea protein has a stronger effect on lowering food intake 30 min later compared to whey protein, egg albumin and maltodextrin. This was further supported through higher feelings of satiety after the casein and pea protein preload. However, this effect on food intake was attenuated when the preload was consumed immediately before the ad libitum meal.

Ad libitum energy intake was lower after the pea protein and the casein preloads in the first experiment and showed a trend in the second experiment. These findings demonstrate pea protein and casein as candidate proteins for satiety. In the literature, there are inconsistent findings related to protein source and satiety when a 45-50 g dose is used. Previous studies have shown similar [9] or lower effect on food intake [7] when whey was compared to casein. These inconsistencies can be attributed to different reasons including dose, study design, subject sample, as well as different physical properties of the proteins used. Even within the same source of protein, attributes can differ with regards to degree of hydrolyzation of the peptides, aggregation of the peptides (micelles) and purity of the isolates used.

Unlike casein protein, pea protein has not been extensively investigated. In the present study, we show for the first time that pea protein is effective in lowering short-term food intake. A few studies have shown either similar or lower food intake after pea protein [12] and pea protein hydrolyzate [14] respectively compared to other proteins. Mechanisms were not explored but gastric emptying might play a role. Casein has been shown to exhibit slower gastric emptying compared to whey protein [7]. Other potential mechanisms can be related to the action of satiety hormones.

When the preload was consumed 30 min before the ad libitum meal, total food intake was similar after casein and pea protein compared to after water control, with a caloric compensation of around 100%. This means that subjects were able to compensate only for the calories in the casein or pea protein preload and not more. Except for a limited number of fiber studies [28,29], it is rare to find an effect of a preload on total food intake that is superior to the calories of the preload. Accordingly, there is limited basis for recommending the ingestion of a preload or a snack to reduce total food intake when simply drinking water or not ingesting the preload or snack results in the same effect. We therefore suggested conducting a second experiment to measure satiation where the preload is given right before the ad libitum meal in order to maximize its effect on the meal or caloric compensation. Contrary to our hypothesis, we found that administering the pea protein or casein preload as a starter before the meal did not lower ad libitum food intake. Furthermore, total intake was higher compared to the water control. In the literature, there are no reported studies on protein source and satiation. The observed lack of effect on satiation was perhaps because the drinks were consumed fast and therefore the volume effect might have overridden any potential functional effect of the protein preload. In both experiments, all treatments were iso-volumetric and subjects were instructed to drink the preloads quickly, as a shot. Previous studies have shown that by shortening the delay between the preload and the ad libitum meal, energy compensation is affected by the volume of the undigested preload and not the type of preload served [30-32]. A slow eating rate has been associated with lower food intake and higher satiety [33-35] perhaps due to longer oro-sensory exposure as well as interaction with the gastrointestinal tract to release of satiety signals. Ratings of satiety in the first experiment were higher after the pea protein and casein preloads compared to other preloads. This might explain the observed lower energy intake. In the second experiment, the ratings were measured only after the ad libitum meal and as expected, they did not differ amongst the preloads.

Postprandial glycaemia was measured as a secondary outcome to investigate if the reported second meal effect of whey protein [16,36] persists when the preload is administered 30 min before the meal. Indeed, whey protein blunted the blood glucose response to the ad libitum meal compared to the other protein preloads and water control. Unlike the literature where a fixed meal was administered, which used a fixed meal, our results were reported after an ad libitum meal and therefore should be considered with caution. We did not investigate the mechanisms responsible for this decrease in blood glucose, but others have shown an increase in plasma insulin concentrations after whey protein which could explain the blunted glucose response [16,36]. Furthermore, the time of ingestion of the ad libitum meal (30 min after the preload) corresponds to the peak insulin response after protein and carbohydrate preloads [7,37]

One limitation in the first study is the palatability of the pea protein preload which was lower than the other preloads. It was not possible to control for palatability in the statistical analysis due to its correlation with energy intake. It is not clear how much of the observed effect on energy intake is driven by palatability. For instance, the casein preload, in the first experiment, had a similar palatability to albumin and whey preloads but still resulted in significantly lower energy intake. Studies by Spitzer et al. [38] and Poppitt et al. [39] found little or no evidence for a reduced food intake after low palatable foods. Furthermore, De graaf et al have shown that palatability has an effect on the intake of the preload itself and not on subsequent food intake and satiety [40].

Other limitations include including only male subjects which raises the question of whether these findings can be applied to women as well, and the single blinded design of the studies which could result in a bias on the results. However for the latter, the person administering the treatments was not the same person preparing them, and given that the drinks were served in opaque and covered cups, the bias is quite limited.

## Conclusion

Different protein sources have distinct metabolic and behavioural effects. Casein and pea proteins show a promising effect on lowering short-term food intake. A beneficial impact on satiation may require a slow rate of consumption but this remains to be tested.

## List of abbreviations used

AUC: area under the curve; BMI: body mass index; CHO: carbohydrate; CSS: combined satiety score; e.g.: for example; g: grams, hrs: hours; i.e.: that is; kcal: kilocalorie; kg: kilogram; m: meter; min: minutes; ml: millilitre; mm: millimetre; PC: personal computer; PFC: prospective food consumption; TFEQ: three factor eating questionnaire; VAS: visual analogue scales; vs: versus; yrs: years.

## Competing interests

All authors were employees of Nestec S.A. during the planning and execution of the studies. Co-author L.K. was working at Nestec. S.A. when the research was conducted.

## Authors' contributions

The authors' responsibilities were as follows: RA, LK and KM contributed to the design of the studies. RA, LK and DB collected the data. RM analyzed the data. RA, LK and KM participated in the discussion of the results. All authors read and approved the final manuscript. This project has been funded by Nestec SA.
